# Phycosphere pH of unicellular nano- and micro- phytoplankton cells and consequences for iron speciation

**DOI:** 10.1038/s41396-022-01280-1

**Published:** 2022-07-07

**Authors:** Fengjie Liu, Martha Gledhill, Qiao-Guo Tan, Kechen Zhu, Qiong Zhang, Pascal Salaün, Alessandro Tagliabue, Yanjun Zhang, Dominik Weiss, Eric P. Achterberg, Yuri Korchev

**Affiliations:** 1 Department of Earth Science and Engineering, Imperial College London, South Kensington Campus, London, SW7 2AZ UK; 2 Marine Biogeochemistry Division, GEOMAR Helmholtz Centre for Ocean Research, 24148 Kiel, Germany; 3 School of Environmental Sciences, University of Liverpool, Liverpool, L69 3GP UK; 4 Key Laboratory of the Coastal and Wetland Ecosystems, Ministry of Education, College of Environment and Ecology, Xiamen University, 361102 Xiamen, China; 5 Department of Earth Sciences, University of Oxford, Oxford, OX1 3AN UK; 6 Department of Ocean Science, Hong Kong University of Science and Technology, Clear Water Bay, Hong Kong, China; 7 Southern Marine Science and Engineering Guangdong Laboratory (Zhuhai), Zhuhai, China; 8 Department of Medicine, Imperial College London, Hammersmith Campus, London, W12 0NN UK; 9 Nano Life Science Institute (WPI-NanoLSI), Kanazawa University, Kakuma-machi, Kanazawa, 920-1192 Japan

**Keywords:** Water microbiology, Microbial biooceanography, Biogeochemistry

## Abstract

Surface ocean pH is declining due to anthropogenic atmospheric CO_2_ uptake with a global decline of ~0.3 possible by 2100. Extracellular pH influences a range of biological processes, including nutrient uptake, calcification and silicification. However, there are poor constraints on how pH levels in the extracellular microenvironment surrounding phytoplankton cells (the phycosphere) differ from bulk seawater. This adds uncertainty to biological impacts of environmental change. Furthermore, previous modelling work suggests that phycosphere pH of small cells is close to bulk seawater, and this has not been experimentally verified. Here we observe under 140 μmol photons·m^−2^·s^−1^ the phycosphere pH of *Chlamydomonas concordia* (5 µm diameter), *Emiliania huxleyi* (5 µm), *Coscinodiscus radiatus* (50 µm) and *C. wailesii* (100 µm) are 0.11 ± 0.07, 0.20 ± 0.09, 0.41 ± 0.04 and 0.15 ± 0.20 (mean ± SD) higher than bulk seawater (pH 8.00), respectively. Thickness of the pH boundary layer of *C. wailesii* increases from 18 ± 4 to 122 ± 17 µm when bulk seawater pH decreases from 8.00 to 7.78. Phycosphere pH is regulated by photosynthesis and extracellular enzymatic transformation of bicarbonate, as well as being influenced by light intensity and seawater pH and buffering capacity. The pH change alters Fe speciation in the phycosphere, and hence Fe availability to phytoplankton is likely better predicted by the phycosphere, rather than bulk seawater. Overall, the precise quantification of chemical conditions in the phycosphere is crucial for assessing the sensitivity of marine phytoplankton to ongoing ocean acidification and Fe limitation in surface oceans.

## Introduction

Marine primary producers including phytoplankton and plants, contribute to about 50% of global primary production [1, 2] and thus play a key role in biogeochemical cycles of carbon and nitrogen [3]. Moreover, calcifying and silicifying algae, like coccolithophorids and diatoms, also modulate the cycles of calcium carbonate and silicon through formation of skeletal material. However, seawater chemistry, including pH and aragonite saturation, is changing at rates not seen in hundreds of thousands of years as a consequence of anthropogenic greenhouse gas emissions [4]. Surface ocean pH is projected to decline by around 0.3 by 2100 as the ocean continues to absorb anthropogenic CO_2_ from the atmosphere [5].

A decrease in bulk seawater pH through ocean inorganic carbon uptake and acidification will alter chemical speciation and bioavailability of dissolved iron (Fe) [6], an essential micro-nutrient limiting phytoplankton growth in >30% of surface oceans [7, 8]. Ocean acidification will also affect calcium carbonate production and may drive distinct malformations of coccolith structures for calcifying phytoplankton species [9]. However, pH conditions in the micro-scale region surrounding phytoplankton cells can differ markedly from ambient bulk seawater, as observed in giant diatoms and algal colonies [10–13]. The microenvironment, known as the phycosphere [14], is the unstirred boundary layer in the immediate vicinity of an algal cell, where the effects of algal metabolisms and other associated microorganisms can be significant.

Currently we lack critical information regarding how pH levels in the phycosphere are controlled for many ecologically important groups of plankton such as unicellular pico-, nano- and micro- phytoplankton. Previous modelling work suggests that the phycosphere pH of these small cells does not significantly differ from bulk seawater [15], due to their small size and the seawater pH buffering capacity. However, this has not been experimentally verified, partly due to a paucity of in situ analytical techniques for the phycosphere pH measurements in those pico- and nano- phytoplankton species. The pH micro-electrodes have been successfully employed for the large diatoms *Odontella sinensis* [10] and *Coscinodiscus wailesii* [12]. To know how those ubiquitous small phytoplankton species will respond to ocean acidification, their phycosphere pH needs to be determined.

Here, via the application of a newly developed pH-sensing nano-probe, originally designed for cancer research and allowing high spatial and temporal resolution quantification (down to 50 nm spatial resolution and 2 ms response time) [16], we aimed to verify that the phycosphere pH of small cells does not differ from bulk seawater. By determining the phycosphere pH of model marine diatoms, green algae and coccolithophores under different environmental conditions, i.e. changing light, seawater pH and buffering capacity, we gain new insights into the underlying mechanisms of phycosphere pH regulation and its responses to ambient environmental changes. We therefore challenge the assumption that phycosphere pH of small cells does not differ from bulk seawater and constrain the impact of changing environmental conditions. Moreover, using newly derived proton and Fe-binding constants for marine dissolved organic matter (DOM) [17, 18], we investigate the influences of the phycosphere on Fe speciation and its availability to phytoplankton.

## Materials and methods

### Model marine phytoplankton species

We used two diatoms (*Coscinodiscus wailesii* CCAP 1013/9, ~100 µm diameter; *C. radiatus* CCAP1013/11, ~50 µm), one green alga (*Chlamydomonas concordia* RCC1, ~5 µm) and one coccolithophore (*Emiliania huxleyi* RCC1731, a calcifying species, ~5 µm). The diatoms were purchased from the Culture Collection of Algae and Protozoa (CCAP) at the Scottish Association for Marine Science while the other two species were from the Roscoff Culture Collection, France. They are non-axenic strains although we maintained them using aseptic techniques, and we cannot exclude the presence of bacteria in the culture.

All species were grown in f/2 medium [19] and in a controlled environmental growth room (fitotron) at 15 °C with an illumination of 110 μmol m^−2^ s^−1^ (16 h light/8 h dark) at Silwood park campus of Imperial College London. Fresh batches were inoculated in a laminar hood. The f/2 medium was prepared using aseptic techniques and laboratory materials were acid-cleaned. Chemicals of ACS grade or higher purity were purchased from Sigma-Aldrich, and artificial seawater and major nutrients were sterilised at 121 °C for 15 min before adding 0.2 µm filtered (polycarbonate filters, Merck Millipore Ltd.) solutions of trace metals and vitamins.

### Phycosphere pH measurements via pH sensing nano-probes coupling with a scanning ion conductance microscopy

**Fig. 1:**
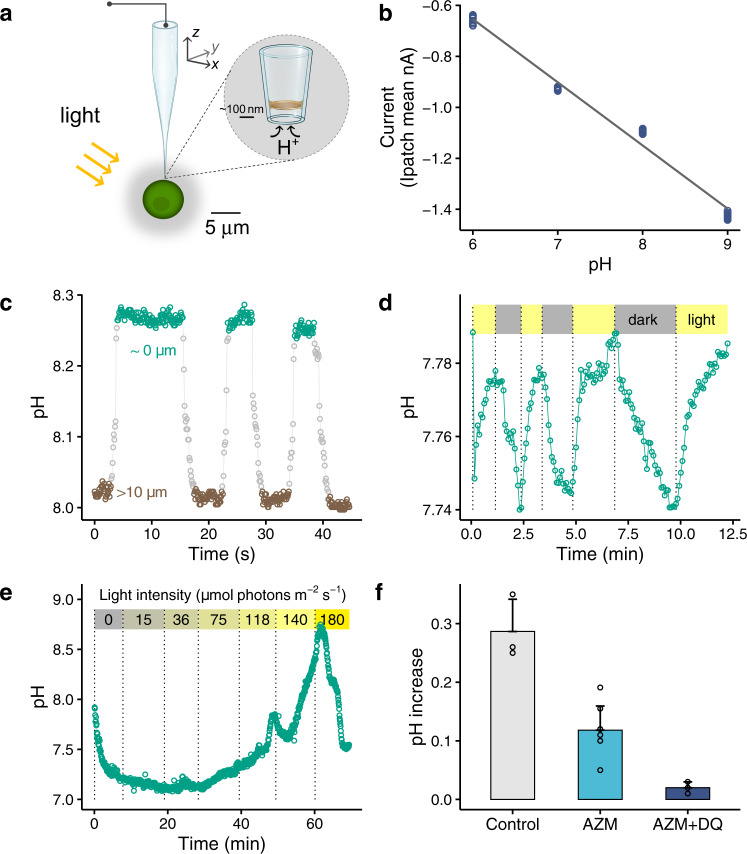
Phycosphere pH of marine green algae *Chlamydomonas concordia* RCC1 (~5 µm diameter). **a** A schematic showing the operation of a pH sensing nano-probe for in situ measurement of pH in the phycosphere of a single cell *C. concordia* by the scanning ion conductance microscopy. **b** The relationship between the measured ion current of the pH nano-probe and seawater pH (*n* = 12, *r*^2^ = 0.99, *p* < 0.0001). **c** A signifi**c**ant pH rising when closing to an illuminated cell. The difference in the pH at the cell surface (8.27 ± 0.01, ~0 µm away from the cell) and that at >10 µm measuring point (8.01 ± 0.01) was significant (*p* < 0.0001). The flagella-mediated motility of *C. concordia* reduced the spatial resolution of local pH profile. Bulk seawater pH = 8.00. **d** A representative pattern for the pH change in the phycosphere under consecutive light/dark cycles. Bulk seawater pH = 7.74 and HCO_3_^−^ = 0.4 mM, the low concentration of HCO_3_^−^ was used to facilitate the measurement of light/dark effect. **e** The pH in the phycosphere incr**e**ased with increasing light intensity, and at the highest light intensity the pH decreased due to possible photosynthesis inhibition. Bulk seawater pH = 7.92, HCO_3_^−^ = 0.4 mM. **f** The increase in the phycosphere pH in illuminated cells was significantly inhibited by 100 μM acetazolamide AZM (inhibitor of external carbonic anhydrase, *n* = 8, *p* = 0.000), and the pH increase was completely suppressed (*n* = 3, *p* = 0.074 in comparison with 0.00) following further addition of 8 μM diquat dibromide DQ (inhibitor of photosystem I). Bulk seawater pH = 7.93 and HCO_3_^−^ = 0.4 mM.

We made the pH sensing nano-probes following the procedures described previously [16] (Supporting Information SI), and used a scanning ion conductance microscopy (SICM) for accurate positioning of the pH nano-probe, allowing extracellular pH of single living cells to be measured at a high temporal and spatial resolution [16] (Fig. 1a). Briefly, each pH nano-probe was firstly calibrated using freshly prepared artificial seawater (i.e. major salts were the same as those in AQUIL [19], and no addition of major and minor nutrients) adjusted to pH 6.00, 7.00, 8.00 and 9.00 by addition of 0.1 M HCl or NaOH, and the pH_NBS_ in each solution was determined using a pH meter (MP 220, Mettler Toledo). The recorded ion currents flowing in the nano-probe were low-pass filtered at 1 kHz and analysed with pClamp 10.3 software (Molecular Devices). The seawater temperature was kept constant (typically varying by <0.1 °C within a 30 min measurement) in an air-conditioned room, which was monitored with a TC-344B Automatic Temperature Controller (Warner Instrument Corporation). We observed a linear relationship between measured ion current of the pH nano-probe and pH in seawater solution in the range of 6.00–9.00 (*r*^2^ = 0.99, *p* < 0.001, Fig. 1b), indicating a good performance of the nano-probe at 0.7 M seawater ionic strength. The precision of the pH measurement was ±0.047 at pH 6.01, ±0.018 at pH 7.01, ±0.023 at pH 7.98 and ±0.035 at pH 8.99, respectively (three times the standard deviation (SD) determined from 7 to 10 measurements). We only employed nano-probes of high pH sensitivity for the measurements of phycosphere pH.

Two or three days after renewal of culture medium, typically at exponential growth stage, one milliliter of algae culture was collected and placed in a Petri dish (35 mm diameter), and cells were allowed to settle in the dish. The diatoms settled within a few minutes, while the smaller cells *C. concordia* and *E. huxleyi* took >10 mins to do so. The upper solution was removed, and the cells were rinsed with the artificial seawater at least three times. The artificial seawater was freshly prepared using aseptic techniques in a clean laminar hood to reduce potential contamination by bacteria. Some *C. concordia* and *E. huxleyi* cells were lost during the washing, but >50 cells per dish remained and these were used for the subsequent pH measurements. The dish containing the rinsed cells was finally filled with 3 mL of artificial seawater of experimental interest and placed above a Nikon TE2000 S inverted microscope. The phycosphere pH measurements were repeated with >3 cell batch cultures grown under the same conditions.

To quantify extracellular pH around a cell, the nano-probe was used as previously described [16, 20] via the SICM and an ICAPPIC controller (IC-UN-001, ICAPPIC Ltd., UK) but with some modifications. First, via applying an external holding voltage of −0.2 V, the nano-probe slowly approached the cell by monitoring ion current through the nano-probe (i.e. the SICM feedback distance control). Once the nano-probe automatically stopped near the cell, the nano-probe was moved laterally until it was pushing the cell at which point it was determined to be at the cell surface. When the nano-probe was in a position of research interest, the holding voltage was removed. Then, the ion current was determined over cyclic voltammograms (pClamp 10.3 software, Molecular Devices). In some tests, a fixed voltage (e.g. 0.6 V or 0.3 V) was applied for a faster measurement of local pH (i.e. each measurement was done in <0.1 s).

We quantified the effect of light intensity, seawater pH and buffering capacity on the phycosphere pH. A Nikon lamp was used (Halogen bulbs, 6 V, 30 W: producing a continuous spectrum of light, from near ultraviolet to deep into the infrared, and the doped quartz of the halogen bulbs blocks UV radiation) as the light source equipped with the microscope, and the light intensity at the position of the pH measurement was quantified using a quantum meter (MQ-500, Apogee Instruments, Inc.). Light shined on cells from above, and the pH nano-probe made from transparent borosilicate glass had little interference on the light. The interference of light intensity on the ion current of the nano-probe was evaluated (Fig. S1). To investigate influence of seawater pH on the phycosphere pH, the seawater pH was adjusted to 7.78, 8.10 or 8.40 by adding 0.1 M NaOH or HCl solution, and the cells were maintained at these different pH for 24 h before the measurement (16 h 110 μmol photons·m^−2^·s^−1^/8 h dark). During this time, no nutrients were added to the seawater. To investigate the effects of seawater buffering capacity on the phycosphere pH, we adjusted the concentration of HCO_3_^−^ and the seawater pH, and the buffering capacity was calculated based upon measured total dissolved inorganic carbon (DIC) and alkalinity in the seawater. The alkalinity and DIC were measured using a Total Alkalinity Titrator AS-ALK2 and a Dissolved Inorganic Carbon Analyser AS-C3 (Apollo SciTech Inc., USA), respectively (Table S1). Except where specified otherwise, the artificial seawater contained 2 mM HCO_3_^−^.

To explore the mechanisms underlying the phycosphere pH change, we monitored changes of the phycosphere pH in *C. concordia* and *C. radiatus* cells following addition of two inhibitors, i.e. 100 μM acetazolamide as an inhibitor of external carbonic anhydrase [10], and 8 μM diquat dibromide as an inhibitor of photosystem I. Both inhibitors were dissolved in diluted dimethyl sulfoxide (DMSO) solution before adding into cell culture (the final concentration of DMSO was 0.1% v/v, which had little effect on the phycosphere pH, Fig. S2).

### Calculations of Fe speciation in the phycosphere

Iron speciation was calculated using the non-ideal competitive adsorption (NICA)—Donnan model [21] in combination with an ion-pairing model within the speciation program ORCHESTRA [22]. The NICA-Donnan model accounts for the intrinsic binding properties of natural organic matter and thus allows for calculation of chemical speciation as a function of pH [17, 21, 23]. The model considers marine DOM as a heterogenous mix of compounds with a bimodial distribution of binding sites that are typically described as carboxylic-like and phenolic-like [18]. We used proton binding parameters from [18] and Fe-binding parameters from [17] in our calculations. The NICA-Donnan model scales binding sites to dissolved organic carbon (DOC) concentration and thus models binding to the many hundreds of thousands of individual compounds that make up the total DOM pool in seawater [24] including algal exudates [25]. However, Fe is also known to be bound to siderophores [26–28], which are produced by bacteria and fungi but not eukaryotic phytoplankton as a high affinity Fe uptake mechanism. Siderophores do not necessarily scale with DOC concentration, we therefore explicitly represent Fe binding to them via addition of equilibrium constants for desferrioxamine B, a well characterised siderophore, to the ion-pairing model [29](Table S2). At this stage we are not able to consider any changes in DOM binding properties in the phycosphere that might result from production of algal/bacterial exudates, since there is very limited information on the impact of the exudates on the overall binding properties of marine organic matter and intrinsic NICA Fe-binding parameters for the exudates are not currently available.

We calculated changes in the fractions of inorganic Fe species, Fe bound to siderophores and Fe bound to DOM as phycosphere pH changed. Two types of seawaters were considered: a “coastal seawater” with 1 nM total dissolved Fe, 5 pM siderophores and 229 µM DOM, and an “open ocean” seawater with 0.1 nM total dissolved Fe, 5 pM siderophores and 57 µM DOM. The selected concentrations of Fe, siderophores, and DOM in bulk seawaters were based on literature values [26, 27, 30, 31]. Since the chemical characteristics of the phycosphere are unknown, we used concentrations of siderophores and DOM in the phycosphere similar to, higher or lower than those in bulk seawaters for the present modelling. In this way, we estimated the impact of any changes in concentrations of DOM and siderophores in the phycosphere in comparison to the bulk media [14, 32].

### Statistical analyses

The SPSS 16.0 software package (SPSS Inc.) was used for the statistical data analysis. Significance of observed changes in phycosphere pH following the different experimental conditions was assessed by using a *t*-test (two-tailed). The figures were processed using the “ggplot2” package in R (V 3.6.1). The phycosphere pH values are expressed as mean ± SD, and the number of observations (n) represent biological replicates except where otherwise stated.

## Results and discussion

### Phycosphere pH of single phytoplankton cells

The pH in the phycosphere of a single cell *Chlamydomonas concordia* (~5 µm diameter) exposed to 140 μmol photons m^−2^ s^−1^ was 8.27 ± 0.01 (179 measurements), while the pH of bulk seawater was 8.01 ± 0.01 (160 measurements) (Fig. 1c). The observed pH variation near the cell surface was <0.03 when the probe was held in place for 45 s. Moving the nano-probe away from the cell resulted in a progressive pH decrease towards the level in the bulk medium, while the pH gradually increased when moving it towards the cell. Measurements on the same cell and different cells were repeated several times, and higher phycosphere pH values than the bulk seawater were consistently recorded (Fig. 1c, Table S3). Under dark conditions, the phycosphere pH decreased, and it increased again once exposed to light (Fig. 1d). Light is thus a major influence on phycosphere pH.

Light intensity controls the magnitude of the phycosphere pH change (Fig. 1e). A gradual increase in light intensity (i.e. 15, 36, 75, 118, 140 and 180 μmol photons m^−2^ s^−1^) progressively increased the phycosphere pH up to 8.71 ± 0.02 (13 measurements), likely due to enhanced inorganic carbon uptake by phytoplankton. In this experiment, the significant change in the phycosphere pH with light was facilitated by the low buffer capacity of the solution (0.4 mM bicarbonate, Table S1); the change was smaller in natural seawater with 2 mM bicarbonate (Table S3). About 2 min later at the highest light intensity of 180 μmol photons m^−2^ s^−1^ the phycosphere pH decreased (Fig. 1e), and this was due to the inhibition of photosynthesis of marine Chlorophyta including *Chlamydomonas* sp. at a high light intensity >150 µmol m^−2^ s^−1^ [33]. At light intensities <118 µmol m^−2^ s^−1^, the phycosphere pH was significantly lower than ambient seawater pH (Fig. 1e), and the decreases in pH likely resulted from weaker photosynthesis, algal respiration, and the possible presence of bacteria in the phycosphere, which would release CO_2_ via respiration. Based upon these observations, we infer that the magnitude of phycosphere pH change in natural phytoplankton assemblages is depth dependent, since photosynthetically active radiation gradually decreases from a few thousands μmol m^−2^ s^−1^ at the surface to <1 μmol m^−2^ s^−1^ at a depth of several hundred metres [34].

We then investigated the mechanisms underlying the regulation of phycosphere pH, and our data indicate that both extracellular and intracellular processes associated with photosynthesis play a role (Fig. 1f). First, the extracellular transformation of bicarbonate by carbonic anhydrase at the cell surface of *C. concordia* and subsequent release of hydroxides contributed to an increase in pH. Specifically, upon addition of 100 μM acetazolamide (inhibitor of external carbonic anhydrase), the increase in the phycosphere pH was significantly reduced from 0.29 ± 0.06 to 0.12 ± 0.04 (*n* = 3–8, *p* = 0.000). Second, we observed no significant increase in the phycosphere pH (0.02 ± 0.01, *n* = 3, *p* = 0.074), upon a further addition of 8 μM diquat dibromide, an inhibitor of photosystem I. Similar responses to the inhibitors were observed in marine diatoms *Coscinodiscus radiatus* (Fig. S3) and the large diatoms *Odontella sinensis* [10].

**Fig. 2:**
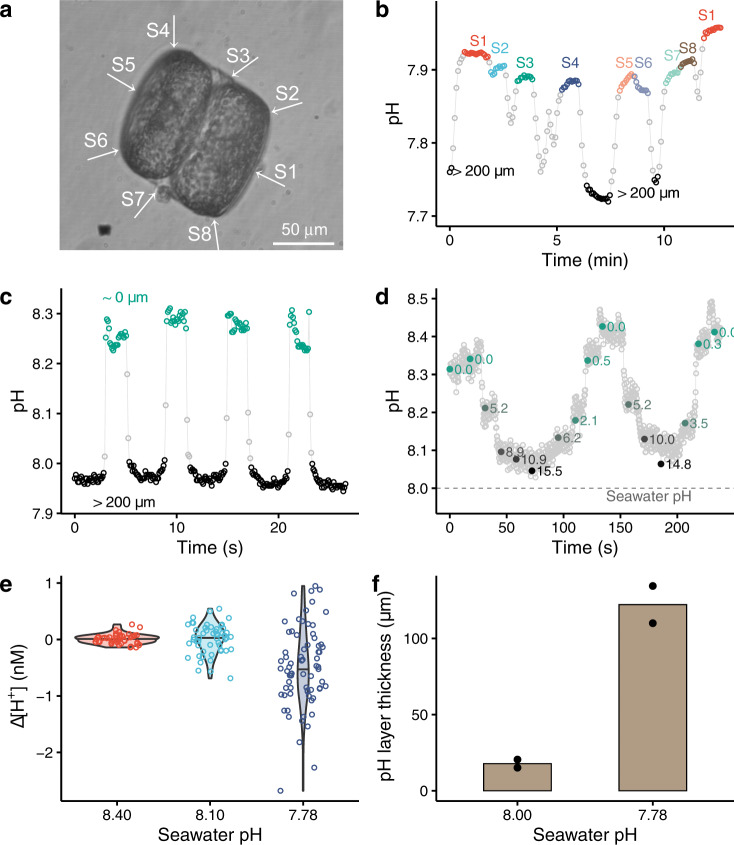
Phycosphere pH of marine diatoms *Coscinodiscus wailesii* CCAP 1013/9 (~50 µm radius). **a** A photo of *C. wailesii* and the arrows showing the measurement positions which correspond to the letters given in the **b**. **b** The measured pH at different surface sites of an illuminated diatom. Bulk seawater pH = 7.76, HCO_3_^−^ = 0.4 mM. **c** The pH changes when the pH probe was approaching or moving away from an illuminated cell. Bulk seawater pH = 7.97. **d** pH at different distances from an illuminated cell. The numbers alongside the dots indicate the distance to the cell (µm). Bulk seawater pH = 8.00. **e** The decrease of H^+^ concentration in the phycosphere (i.e. pH increase) at seawater pH 7.78 was significantly higher than that at bulk pH = 8.10 (*n* = 53–71, *p* = 0.000) while the difference in ΔH^+^ of the phycosphere between seawater 8.40 and 8.10 was insignificant (*n* = 47–53, *p* = 0.920). **f** The thickness of the pH boundary layer at seawater pH 7.78 was significantly larger than that at bulk pH 8.00 (*n* = 2, *p* = 0.014).

Detailed phycosphere pH measurements with the diatom *Coscinodiscus wailesii* of ~50 µm radius were undertaken. We firstly determined pH at 8 positions along the surface of an illuminated cell to assess whether pH was uniform at the cell surface (Fig. 2a); the difference among the measured pH at the 8 positions was <0.02 (Fig. 2b). Such a small variation might link to the evenly distributed chloroplasts around this centric diatom [35]. In contrast, for the rod-shaped diatom *O. sinensis*, the phycosphere pH is 0.1 higher in the central region than at the tip of the cell, although the chloroplasts are evenly distributed along the length of the cell [10].

Similar to *C. concordia*, pH increases of 0.30 were observed when moving the pH nano-probe from the bulk medium (pH 7.97 ± 0.01, 166 measurements) to the surface of a *C. wailesii* cell (8.27 ± 0.02, 77 measurements) (Fig. 2c). The thickness of the pH boundary layer, defined as the distance from the cell surface to a position where the measured pH is 99–101% of the bulk seawater, was ~15 µm for *C. wailesii* during light exposure (Fig. 2d). We determined the phycosphere pH in stagnant seawater, but it may vary in naturally turbulent seawater. However, theoretical models suggest that turbulence can only have a significant effect on the unstirred boundary layer in microorganisms of >100 µm in diameter [36], but not in those smaller cells [37].

We found that the phycosphere pH (i.e. H^+^ concentration) in *C. wailesii* was sensitive to the pH of the bulk seawater (Fig. 2e). The increase in phycosphere pH (i.e. the decrease in H^+^ concentration) of diatoms exposed to seawater of a lower pH (i.e. pH 7.78) was significantly higher than those exposed to seawater of higher pH (i.e. pH 8.10 or 8.40) (*p* = 0.000). Similarly, the giant marine diatom *O. sinensis* experiences much greater pH increases within the phycosphere at bulk seawater pH 7.60 than pH 8.20 [10]. The experimental observations generally agree with the previous modelling work which predicts that the difference in pH between phycosphere and bulk seawater will increase in future as the buffering capacity of seawater decreases [15]. This is in line with the buffering capacity of our seawater solutions decreasing with decrease of the seawater pH (Table S1).

In addition to the seawater buffering capacity, our data indicate that the biological processes responsible for the phycosphere pH are sensitive to ambient seawater pH. Specifically, if there were no changes in such biological processes, we would have seen a bigger decrease in the phycosphere H^+^ concentration at pH 8.10 than at pH 8.40, in agreement with the reduced buffering capacity at the lower pH. But we observed no significant difference in average phycosphere H^+^ shift between the diatoms exposed to seawater of pH 8.40 and 8.10 (Fig. 2e).

The nutritional status of algae cells also plays an important role in the magnitude of the phycosphere pH change. Following a 24 h starvation of nutrients (i.e. N, P, Si and micronutrients), the measured changes in the phycosphere pH of *C. wailesii* cells exposed to different seawater pH were consistently smaller than the nutrient-replete cells (Fig. 2e versus Fig. S8). Similarly, a previous study reports a higher pH in the phycosphere of Fe-replete diatoms *Thalassiosira weissflogii* than Fe-limited cells [11]. Overall, the limitation or starvation by nutrients would have decreased photosynthesis and/or extracellular carbonic anhydrase of these diatoms, and hence reduced the overall phycosphere pH change.

The thickness of the pH boundary layer is sensitive to ambient bulk seawater pH. For *C. wailesii* in the light, the thickness of the layer at bulk seawater pH 7.78 was 122 ± 17 µm, which was sevenfold thicker than that at a bulk seawater pH of 8.00 (18 ± 4 µm, *p* = 0.014, Fig. 2f). The large increase in thickness of the layer at a lower bulk seawater pH arose from the smaller buffering capacity of the exposure solution (Table S1); the H^+^ would travel a longer distance in seawater of a lower pH buffering capacity, leading to a thicker diffusive boundary layer around a cell. When bulk seawater was fixed at pH 8.00, but buffering capacity reduced by altering the bicarbonate concentration, we found the thickness of phycosphere pH layer in diatoms increased (Fig. S4), consistent with the longer transport distance calculated for CO_2_ in seawater with a lower pH and buffering capacity [38]. Hence, we show that both seawater pH and buffering capacity play an important role in setting the phycosphere thickness.

**Fig. 3:**
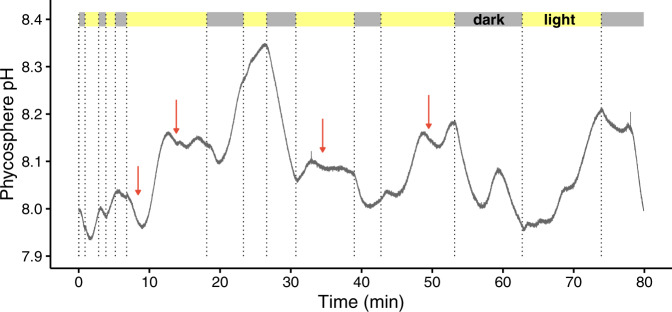
Phycosphere pH of marine coccolithophore *Emiliania huxleyi* in the light (140 μmol photons m^−2^ s^−1^) and in the dark. Bulk seawater pH = 8.00. Note, in the light the phycosphere pH did not always increase and sometimes decreased (red arrows), and such decreases likely result from biogenic calcification, which releases protons or CO_2_.

Increases of the phycosphere pH were observed in the coccolithophore *Emiliania huxleyi* upon exposure to 140 μmol photons m^−2^ s^−1^ (Fig. 3). However, the phycosphere pH of *E. huxleyi* did not always increase, and decreases were also observed in the light. We suspect the decrease of local pH in the light likely resulted from biogenic calcification of this species, as CO_2_ or protons are released in the course of biomineralisation [2]. Indeed, a gradual reduction in pH was observed during foraminiferal calcification in the microenvironment surrounding a calcifying specimen of *Ammonia* sp [39]. Hence, we suspect that the overall pH change in the phycosphere of coccolithophores should be a combined effect of photosynthesis, calcification and respiration; a significant increase in pH in the phycosphere would only be observed when their photosynthesis is stronger than calcification and respiration. Further experimental and modelling work [38] on the interactions between biogenic calcification and phycosphere pH is required.

Our observations on the nano- and micro- phytoplankton species are consistent with those on giant phytoplankton species and algal colonies [10, 12, 40]. Overall, in the seawater of 2 mM bicarbonate at pH 8.0 under 140 μmol m^−2^ s^−1^ light exposure, we observed that the phycosphere pH significantly increased by 0.15 ± 0.20 for *C. wailesii* (*n* = 32, *p* = 0.000), 0.11 ± 0.07 for *C. concordia* (*n* = 7, *p* = 0.005), 0.41 ± 0.04 for *C. radiatus* (*n* = 3, *p* = 0.002) and 0.20 ± 0.09 for *E. huxleyi* (*n* = 5, *p* = 0.008) (Table S3). Moreover, we found that there was a clear variation of the phycosphere pH even within a population of cells. For instance, amongst the 32 individual cells of *C. wailesii*, the phycosphere pH of one cell was 1.12 higher than the bulk seawater while the pH of two cells was not higher than the bulk seawater (*data sheet of* Table S3, 10.6084/m9.figshare.19576477.v1). Such inter-individual differences would be due to their differences in photosynthesis, respiration and/or carbonic anhydrase activity.

### Consequences of phycosphere pH change for Fe speciation and bioavailability

Iron availability to phytoplankton is influenced by seawater chemistry and cell physiology [6, 41, 42]. However, most experiments have not assessed the influence of the phycosphere on Fe speciation and bioavailability. No analytical technique is currently available for direct measurements of Fe speciation in the phycosphere, and even modelling Fe speciation as a function of pH in seawater was previously challenging due to a lack of intrinsic chemical binding parameters for marine DOM [23].

**Fig. 4:**
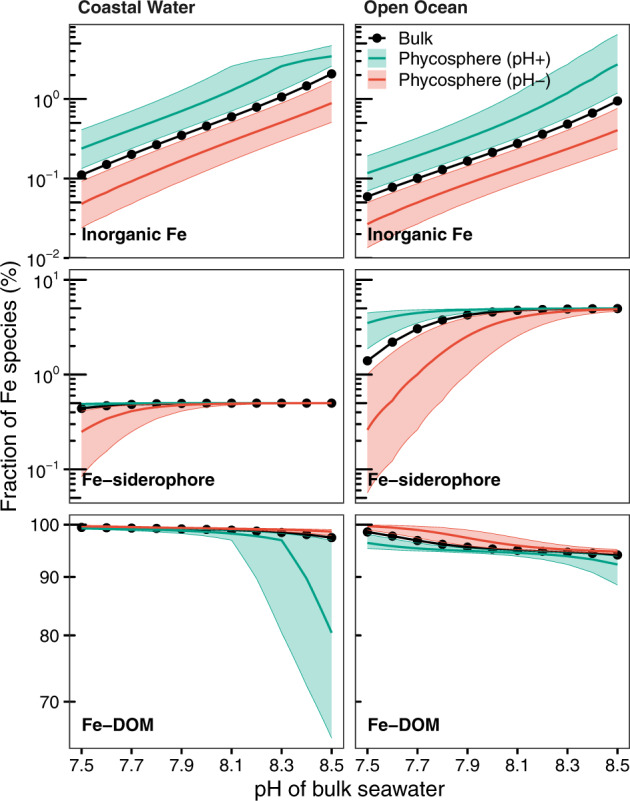
The influence of 0.26 ± 0.20 pH increases/decreases in the phycosphere on the fraction of Fe species (i.e. inorganic Fe species, Fe bound to siderophores and dissolved organic matter DOM). The green/red lines indicate the average changes in the phycosphere, while the shaded areas show their variations. The black lines represent for bulk seawater. The “coastal water” scenario has 1 nM total dissolved Fe, 5 pM siderophores and 229 µM DOM, while the “open ocean” scenario has 0.1 nM total dissolved Fe, 5 pM siderophores and 57 µM DOM. Here, the concentrations of total dissolved Fe, siderophores and DOM are assumed to be the same as those in bulk seawater. Changes in Fe speciation in the phycosphere produced by increased/decreased concentrations of H^+^, DOM and siderophores in the phycosphere relative to bulk seawater are shown in the supporting information.

Here, we took advantage of newly derived proton and Fe-binding parameters for marine DOM [17, 18], and calculated the effect of a 0.26 pH change in the phycosphere on Fe speciation for phytoplankton living in coastal and open ocean environments (Fig. 4). For both scenarios, when the phycosphere pH increases by 0.26, the fraction of inorganic Fe species, which is considered to be directly available for biological uptake [43], increases by ~2 fold. These increases arise because, with the organic matter binding parameters predicted with our model, the hydroxide ion (OH^−^) competes more effectively for Fe as pH increases. On the other hand, the fraction of inorganic Fe species decreases by 50% when the phycosphere pH decreases by 0.26. Such changes in inorganic Fe species are not trivial and might have significant impacts on Fe bioavailability and growth of marine phytoplankton, because even dissociation of 2% of organic Fe complexes can markedly improve the growth of many oceanic algae species [44].

In addition to pH, the concentrations of DOM, siderophores and other Fe-binding ligands in the phycosphere might differ from bulk seawater as a result of algae and associated bacteria metabolism [14, 32]; under such scenarios our calculations show that the phycosphere Fe speciation is largely different from that in bulk seawater (Figs. S5 and S6). For instance, when the concentrations of DOM and siderophores increase by tenfold in this microenvironment as a result of intensive algal/bacterial exudation (Fig. S6), the fraction of inorganic Fe species in the phycosphere becomes negligible (<0.01% of total dissolved Fe) and is 100-fold lower than the bulk seawater. Further increases in pH in the phycosphere results in a higher proportion of Fe bound to siderophore but less binding to DOM. On the other hand, when the local concentrations of DOM and siderophores decrease by tenfold via e.g. bacterial consumption, some dissolved Fe precipitates as Fe(OH)_3_ and a higher pH in the phycosphere then leads to the formation of more Fe precipitates (Fig. S5).

**Fig. 5:**
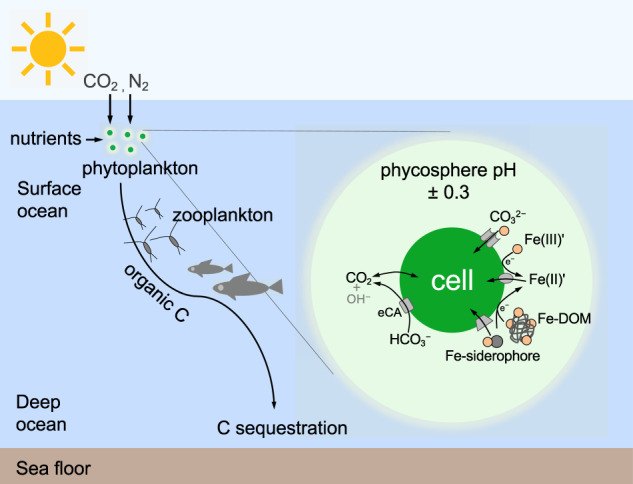
Phycosphere pH and consequences for local Fe speciation and its availability to phytoplankton. Possible impact of an increase of pH in the phycosphere driven by algal uptake of CO_2_ and extracellular enzymatic transformation of HCO_3_^−^ and a pH decrease driven by CO_2_ release from the phytoplankton cell or associated bacteria on Fe speciation and its availability to marine phytoplankton, which influence biogeochemical cycles of C, N, Si, and trace elements in the oceans. Specifically, Fe bioavailability may be significantly altered, as concentrations of inorganic Fe species (Fe(III)’), carbonate-coordinated Fe(III) uptake and/or organic Fe complexes (Fe-DOM and Fe-siderophores) change in the phycosphere. eCA extracellular carbonic anhydrase.

Based on our results, we propose that the pH change in the phycosphere alters Fe availability to phytoplankton. An increase in the phycosphere pH enhances Fe bioavailability via three pathways (Fig. 5): (a) a higher pH in the phycosphere increases the abundance of inorganic Fe species and hence Fe bioavailability; (b) a higher local pH increases the availability of carbonate in the phycosphere and hence will facilitate Fe(III) uptake by carbonate sensitive transferrins in certain diatoms [45]; and (c) elevated phycosphere pH increases the amount of algal surface-bound Fe [46] and hence facilitates Fe bio-uptake. We suggest that the light-induced increase in pH in the phycosphere is likely an important component of Fe acquisition in phytoplankton.

In contrast to the effect of the pH increase in the phycosphere, dark and low light intensities likely reduce Fe availability to phytoplankton cells. This arises because a decrease in the phycosphere pH decreases the concentration of inorganic Fe species, carbonate and surface bound Fe. In addition, altered abundance/chemistry of Fe-binding ligands in the microenvironment as a consequence of algal/bacterial metabolisms could further change the Fe speciation and hence Fe bioavailability. At present, very few studies have investigated the influences of organic ligands and bacteria in the phycosphere on Fe bioavailability; one study [47] shows that an algal-associated bacterium *Marinobacter* sp. increases the Fe uptake by 70% and dinoflagellate partner *Scrippsiella trochoidea* by >20-fold with a light radiation of 450 μmol m^−2^ s^−1^.

This study shows that even in the cells of ~5 µm diameter, the pH in the phycosphere is consistently different from bulk seawater. For the first time, our data show that the thickness of the pH boundary layer is largely amplified by ocean acidification. Moreover, our modelling results suggest that the local pH alters Fe speciation in this microenvironment, and in a future more acidic ocean, a much thicker boundary layer will result in a larger deviation of the Fe speciation in the phycosphere from bulk seawater. In addition, we suspect that the local pH microenvironment would influence biogenic calcification; for instance, higher phycosphere pH likely favours extracellular precipitation of CaCO_3_ in certain holococcolith-forming species such as *Coccolithus pelagicus* and *Calyptrosphaera sphaeroidea* [48].

Precise quantification of chemical conditions in the phycosphere is crucial for better understanding how phytoplankton will respond to environmental changes. Evidence is emerging that interactions between phytoplankton and abiotic/biotic environments are governed by micro- and nano- scale interfacial processes [13, 47, 49, 50], which cannot be determined using bulk water analyses. Small changes in the phycosphere likely translate into large impacts on the oceanic carbon and nitrogen cycle (Fig. 5). Even a minor increase in Fe availability could result in a large amount of biological CO_2_ and N_2_ fixation, on the order of 400,000 atoms of C and/or 60,000 atoms of N per Fe atom [44, 51].

## Supplementary information

Supporting information

## Data Availability

The datasets analysed during the current study are available in the Figshare repository via the link 10.6084/m9.figshare.19576477.v1. ORCHESTRA and MINTEQ4 database can be downloaded from http://orchestra.meeussen.nl/downloads/. A protocol giving instructions on how to use ORCHESTRA to calculate iron speciation in seawater is accessible on protocols.io via the link 10.17504/protocols.io.brc4m2yw.
