# Supplementary material for: Phycosphere pH of unicellular nano- and micro- phytoplankton cells and consequences for iron speciation
Source: ISME J. 2022 Jul 7;16(10):2329–36. doi: 10.1038/s41396-022-01280-1 (PMC9478132; doi:10.1038/s41396-022-01280-1)
Supplement: Supplementary file 1 — Supporting information [file 41396_2022_1280_MOESM1_ESM.pdf]

Supporting Information

Phycosphere pH of unicellular nano- and micro- phytoplankton cells and consequences for iron speciation

Fengjie Liu[1,2,3]*, Martha Gledhill[2], Qiao-Guo Tan[4], Kechen Zhu[2], Qiong Zhang[5,6,7], Pascal Salaun[3], Alessandro Tagliabue[3], Yanjun Zhang[8,9]*, Dominik Weiss[1]*, Eric P. Achterberg[2], Yuri Korchev[8,9]*

[1]Department of Earth Science and Engineering, Imperial College London, South Kensington Campus, London, SW7 2AZ, United Kingdom

[2]Marine Biogeochemistry Division, GEOMAR Helmholtz Centre for Ocean Research, Kiel, 24148, Germany

[3]School of Environmental Sciences, University of Liverpool, Liverpool, L69 3GP, United Kingdom

[4]Key Laboratory of the Coastal and Wetland Ecosystems, Ministry of Education, College of Environment and Ecology, Xiamen University, Xiamen, 361102, China

[5]Department of Earth Sciences, University of Oxford, Oxford, OX1 3AN, United Kingdom

[6]Department of Ocean Science, Hong Kong University of Science and Technology, Clear Water Bay, Hong Kong, China

[7]Southern Marine Science and Engineering Guangdong Laboratory (Zhuhai), Zhuhai, China

[8]Department of Medicine, Imperial College London, Hammersmith Campus, London, W12 0NN, United Kingdom

[9]Nano Life Science Institute (WPI-NanoLSI), Kanazawa University, Kakuma-machi, Kanazawa, 920-1192, Japan

*Corresponding authors: fliu@geomar.de, yanjun.zhang@imperial.ac.uk, d.weiss@imperial.ac.uk, y.korchev@imperial.ac.uk

Contents

Note S1. Fabrication of pH sensing nano-probes and our customised SICM setup……………….3

Figure S1. The potential interference of light on the ion current of the pH nano-probe……….4

Figure S2. The potential influence of dimethyl sulfoxide DMSO on the phycosphere pH………4

Figure S3. The phycosphere pH in diatoms *Coscinodiscus radiatus* following the addition of acetazolamide and diquat dibromide…………………………………………………………………………………5

Figure S4. The thickness of pH boundary layer in diatoms *C. wailesii* under different concentrations of bicarbonate……………………………………………………………………………………………5

Figure S5. The influence of pH change in the phycosphere on the Fe speciation when the concentrations of siderophores and DOM in the phycosphere are assumed to be 10% of those in bulk seawater………………………………………………………………………………………………………6

Figure S6. The influence of pH change in the phycosphere on the Fe speciation when the concentrations of siderophores and DOM in the phycosphere are assumed to be 10-fold higher than those in bulk seawater……………………………………………………………………………………7

Figure S7. The signal drift of the pH nano-probe over time in seawater……………………………….8

Figure S8. The phycosphere pH in diatoms *C. wailesii* under different concentrations of bicarbonate……8

Table S1. The carbonate chemistry of our artificial seawater media……………………………………9

Table S2. Parameters of the NICA-Donnan model used for the calculation of the Fe speciation………9

Table S3. A summary of the phycosphere pH in a diversity of phytoplankton species………..10

References………11

Note S1. Fabrication of pH sensing nano-probes and our customised SICM setup

We made the nano-probes by pulling borosilicate glass capillaries (O.D. 1 mm, I.D. 0.5 mm) to nano-pipettes of ~100 nm tip radius using a laser-based puller (Model P-2000, Sutter Instruments Co., USA). Nanopipettes were pulled in two steps as described previously (Zhang et al. 2019): for the first step, the parameters were set as heat 370, filament 3, velocity 32, delay 180 and pull 0; for the second step, they were heat 350, filament 2, velocity 27, delay 130 and pull 50.

The pH-sensing nanoprobes were fabricated by crosslinking negative-charged glucose oxidase (GOx) and positive-charged poly-L-lysine (PLL) to form a pH-sensitive zwitterion-like nanomembrane at the tip of glass nanopipettes (Zhang et al. 2019). Briefly, each nano-pipette was firstly filled by capillary action with a solution of 0.4 mg mL^{-1} GOx dissolved in 0.01% (v/v) PLL. Then, the nano-pipettes were placed into 25% (v/v) glutaraldehyde vapour at room temperature for 48 hours to induce cross-linking reaction between PLL and GOx at the tip of the nano-pipette. Finally, the nano-probes were washed with 0.2 µm-filtered 0.1 M KCl (pH ~7.00) to remove unreacted glutaraldehyde and other un-crosslinked chemicals.

Before the pH calibration in seawater, each pH nano-probe was first backfilled with 0.2-µm filtered 0.1 M KCl (pH ~7.0) and contacted with a measuring Ag/AgCl electrode, then immersed into a bath of 0.3 mL the artificial seawater. Another Ag/AgCl reference electrode was placed in the seawater bath, and all measurements were quoted against this reference electrode. Both electrodes were connected to a MultiClamp 700B amplifier and digitised with an Axon Digidata 1322A and Clampex 9.2 (Molecular Devices, UK). The potential was typically cycled between - 0.6 V and + 0.6 V (or - 0.3 V and + 0.3 V) at a scan rate of 650 mV s^{-1}.

The SICM consisted of a PIHera P-621.2 XY Nano-positioning Stage (Physik Instrumente, Germany) with 100 x 100 µm travel range and a LISA piezo actuator P-753.21C (Physik Instrumente, Germany) with 25 µm travel range for a pH nano-pipette positioning along Z-axis. Coarse positioning was achieved with translational stages M-111.2DG (XY directions) and M-112.1DG (Z-axis) (Physik Instrumente, Germany). The piezo actuator was powered by high voltage amplifiers E-503 and E-505 and servo module E-509 (Physik Instrumente, Germany). Software for the SICM control, data acquisition and analyses were written and kindly provided by Dr. Pavel Novak, ICAPPIC Ltd. The ion current and output of the capacitive sensors from all three piezo elements were monitored using an Axon Digidata 1322A digitiser and Clampex 9.2 software (Molecular Devices, UK).

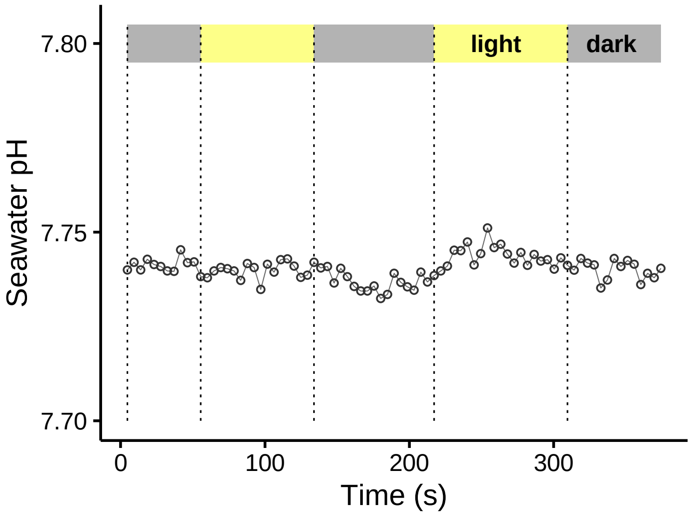

Figure S1. The control experiment in bulk seawater solution showed little response of the pH nano-probe to a change in light intensity (and/or light induced change of seawater temperature), indicating minimal interferences to the pH nano-probe under conditions of our experiment. The grey zone indicates dark conditions, while the yellow zone indicates a light intensity of 140 µmol photons m^{-2} s^{-1}.

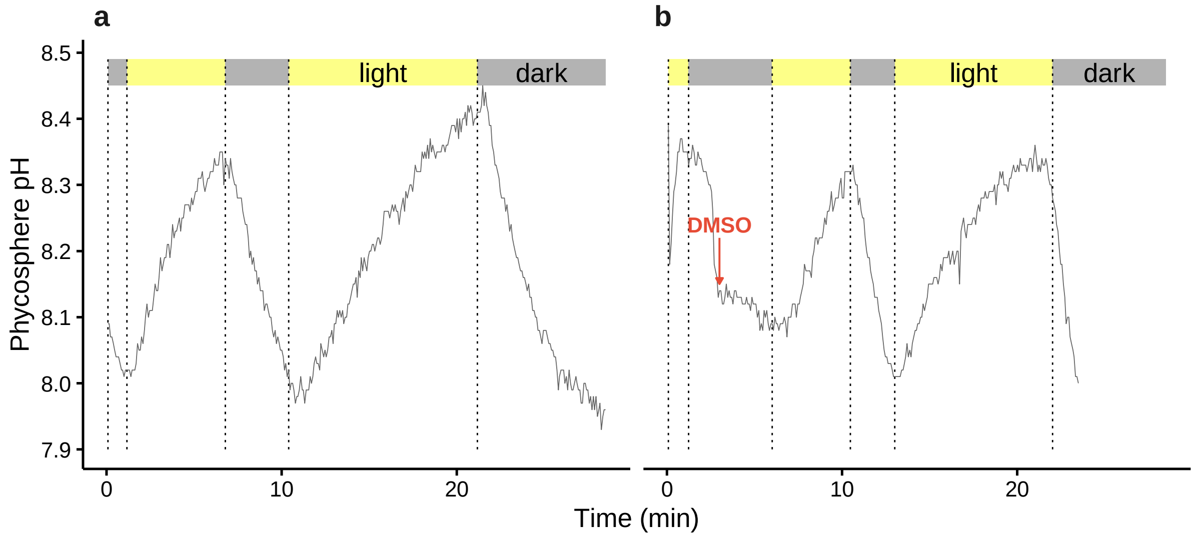

Figure S2. The phycosphere pH change in diatoms *Coscinodiscus radiatus* CCAP1013/11 in the absence (a) or presence (b) of 0.1% dimethyl sulfoxide DMSO v/v, which was used as a solvent for acetazolamide and diquat dibromide. The light intensity = 140 µmol photons m^{-2} s^{-1} and bulk seawater pH = 8.00.

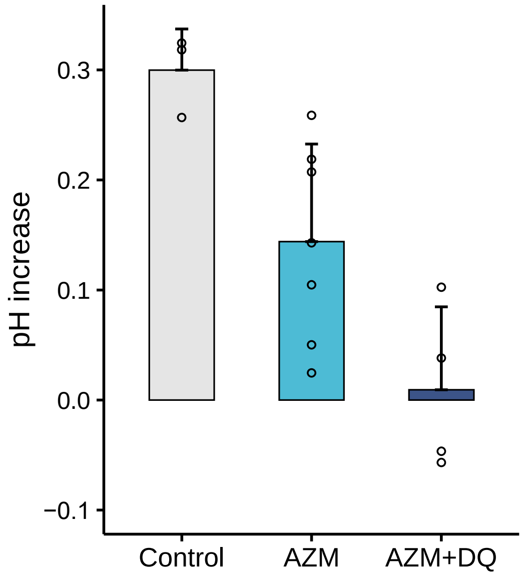

Figure S3. The pH increase in the phycosphere of diatoms *Coscinodiscus radiatus* CCAP1013/11 was significantly inhibited by 100 µM acetazolamide AZM (inhibitor of external carbonic anhydrase) (*t*-test, 2-tailed, $p = 0.022$), and the pH increase became insignificant (*t*-test, 2-tailed, $p = 0.857$, test value = 0) following further addition of 8 µM diquat dibromide DQ (inhibitor of photosystem I) (i.e., in the presence of AZM and DQ. Bars are mean ± SD, $n = 3\text{-}7$. The light intensity = 140 µmol photons m^{-2} s^{-1} and bulk seawater pH = 7.96 buffered by 1 mM HCO_3^-.

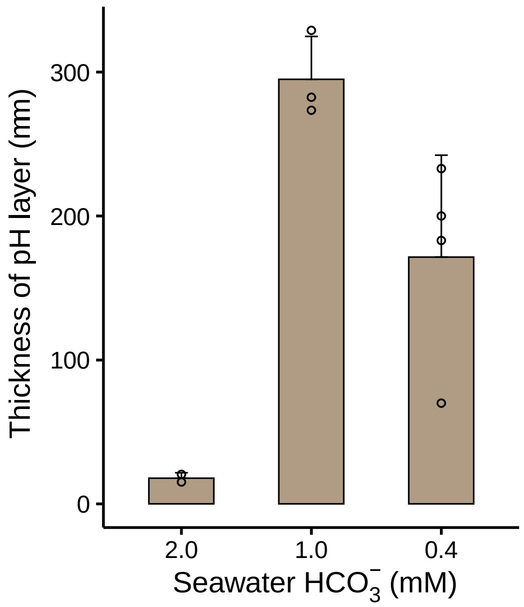

Figure S4. The measured thickness of pH boundary layer in illuminated diatoms *C. wailesii* under different concentrations of bicarbonate (bulk seawater pH = 8.00 and the light intensity = 140 µmol photons m^{-2} s^{-1}). Bars are mean ± SD, $n = 2\text{-}4$. The pH layer was much thicker at 1.0 mM HCO_3^- than that at 2.0 mM HCO_3^- ($p = 0.001$), and so was at 0.4 mM HCO_3^- ($p = 0.044$).

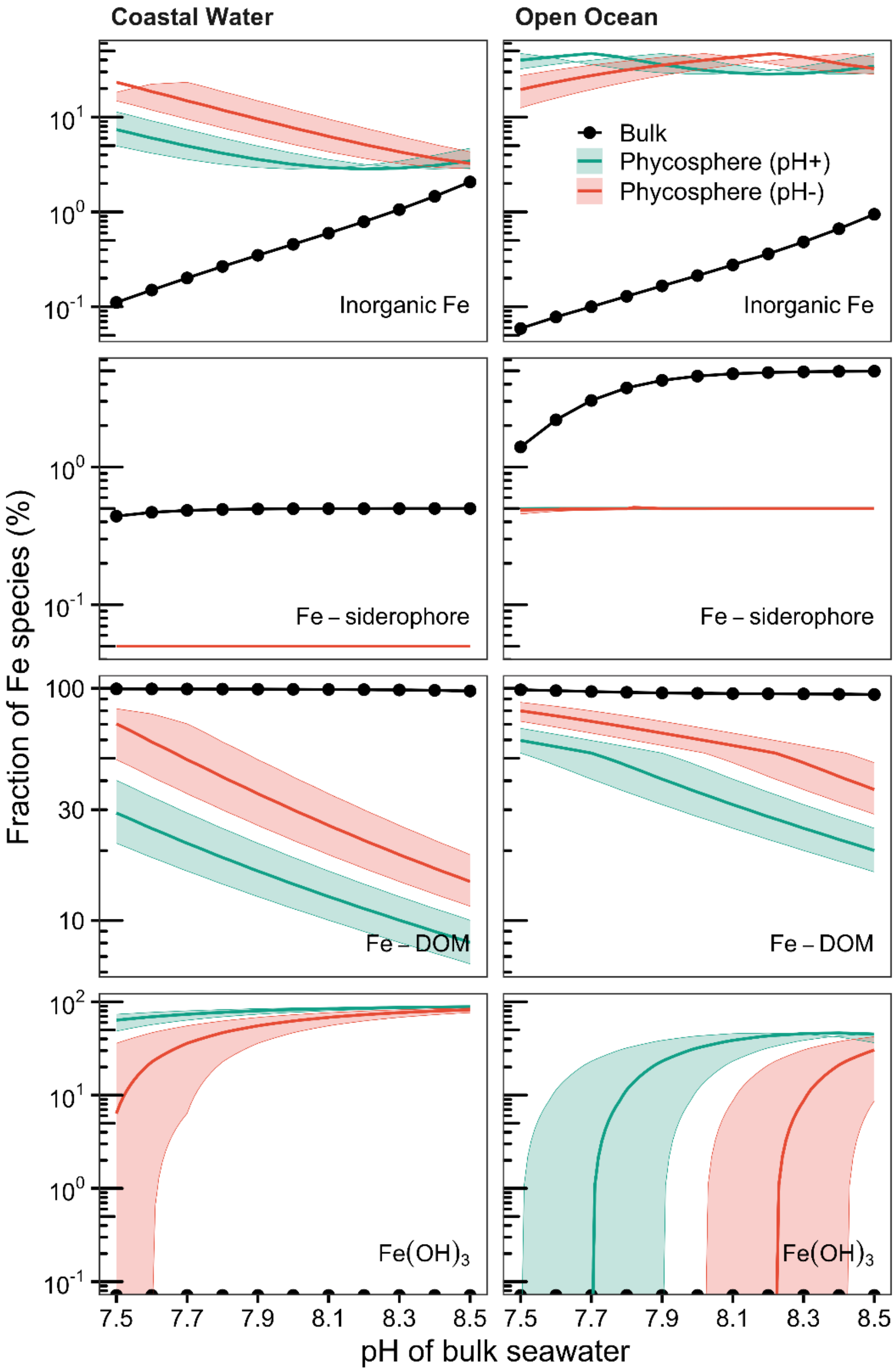

Figure S5. The influence of 0.26 ± 0.20 pH increases/decreases in the phycosphere on the fraction of Fe species (i.e., inorganic Fe species, Fe bound to siderophores, Fe bound to DOM and solid $Fe(OH)_3$). The green/red lines indicate the average changes in the phycosphere, while the shaded areas show their variations. The black lines represent for bulk seawater. The ''coastal water'' scenario has 1 nM total dissolved Fe, 5 pM siderophores and 229 µM dissolved organic matter DOM, while the ''open ocean'' scenario has 0.1 nM total dissolved Fe, 5 pM siderophores and 57 µM DOM. Here, the concentrations of siderophores and DOM in the phycosphere are assumed to be 10% of those in bulk seawater.

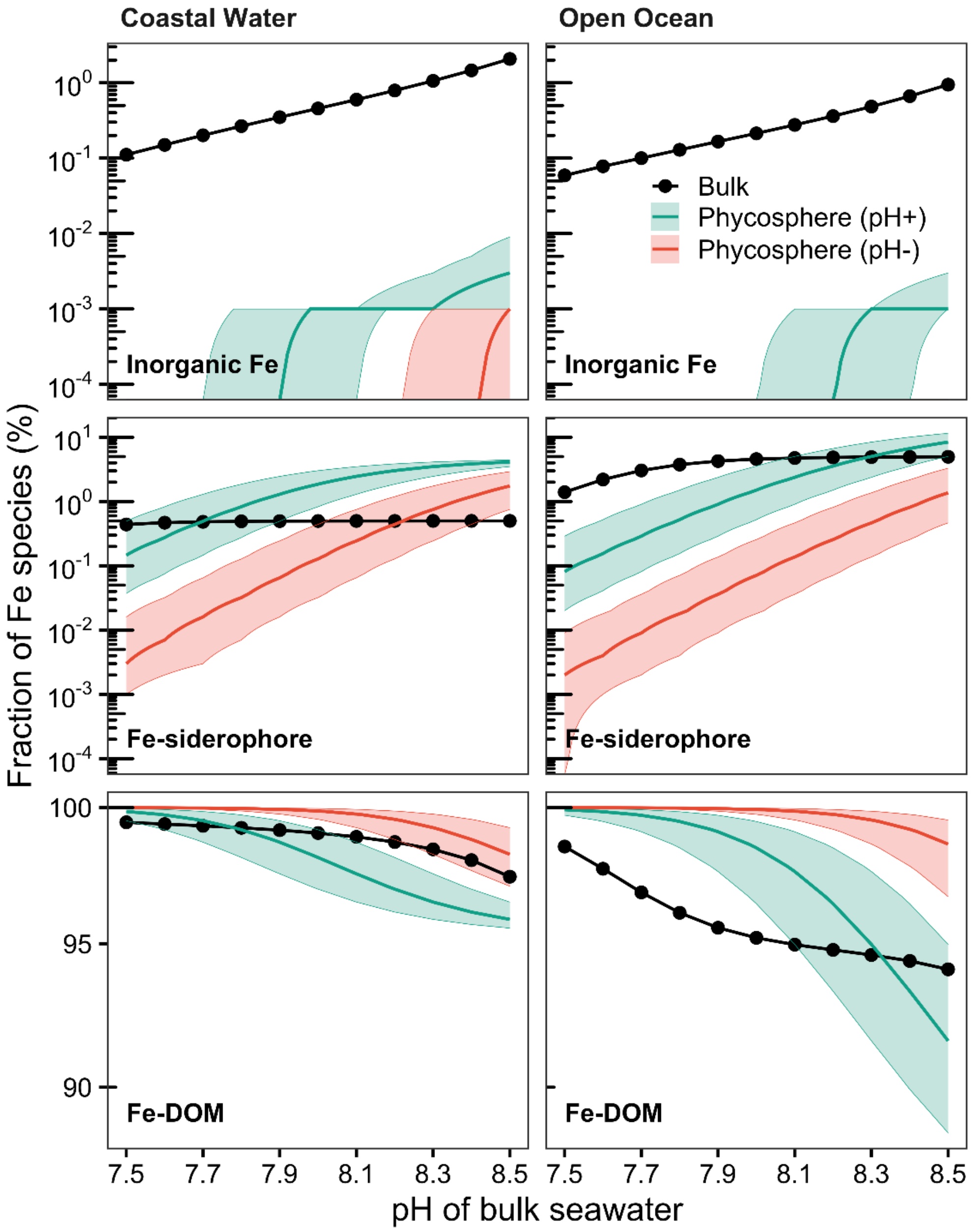

Figure S6. The influence of 0.26 ± 0.20 pH increases/decreases in the phycosphere on the fraction of Fe species (i.e., inorganic Fe species, Fe bound to siderophores and DOM). The green/red lines indicate the average changes in the phycosphere, while the shaded areas show their variations. The ''coastal water'' scenario has 1 nM total dissolved Fe, 5 pM siderophores and 229 µM dissolved organic matter DOM, while the ''open ocean'' scenario has 0.1 nM total dissolved Fe, 5 pM siderophores and 57 µM DOM. Here, the concentrations of siderophores and DOM in the phycosphere are assumed to be 10-fold higher than those in bulk seawater. The modelling indicates no formation of solid $Fe(OH)_3$.

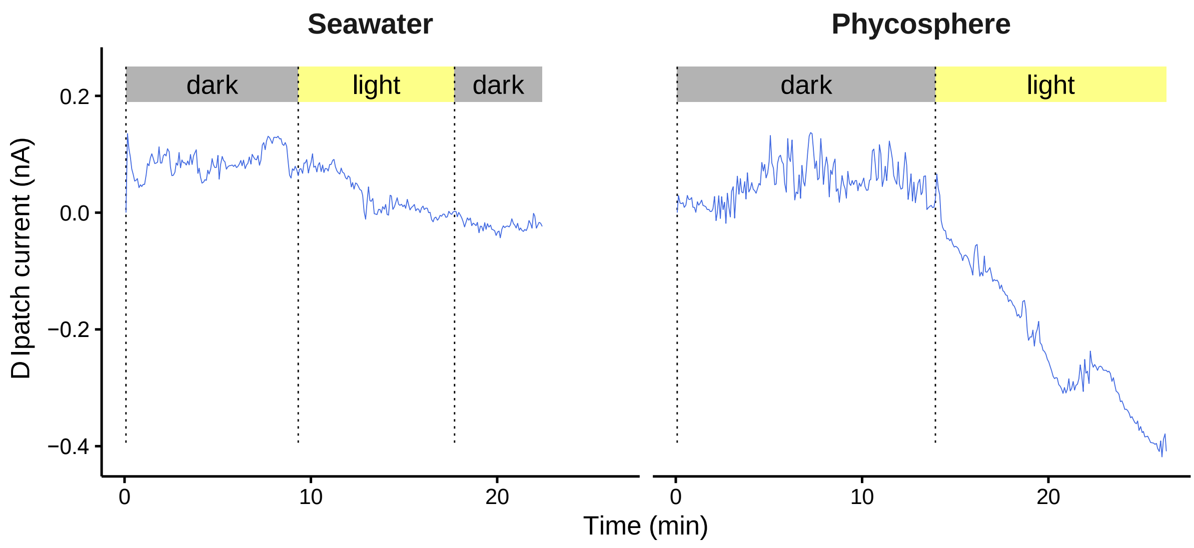

Figure S7. The control experiment showed the variation in the measured mean current of the pH nano-probe in artificial seawater (pH 7.92, 0.4 mM HCO_3^-) and in the phycosphere over >20 minutes in the light (the yellow zone, 140 µmol photons m^{-2} s^{-1}) or under dark (the grey zone). There was a signal drift over time in the light, and the drift rate was 20% of the signal change in the phycosphere.

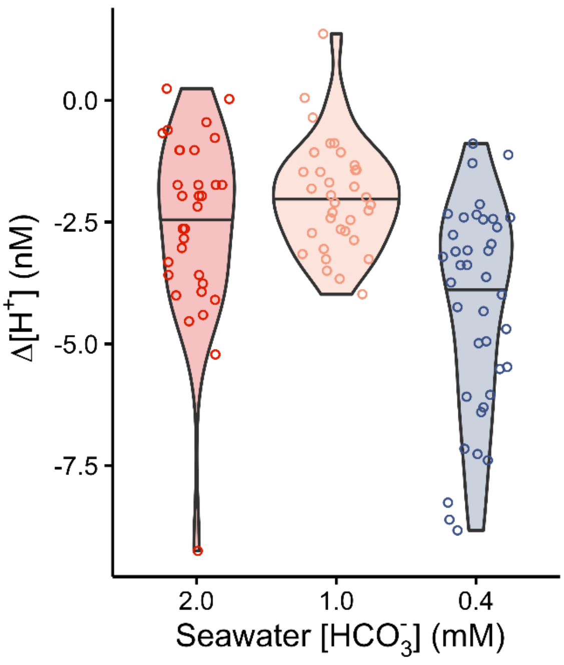

Figure S8. The phycosphere H^+ decrease (i.e., the pH increase) in illuminated diatoms *Coscinodiscus wailesii* at 0.4 mM HCO_3^- was significantly higher than that at 1.0 mM or 2.0 mM HCO_3^- (p = 0.000). Lines are means of n = 32-39 cells. Bulk seawater pH = 8.00 and the light intensity = 140 µmol photons m^{-2} s^{-1}.

Table S1. The carbonate chemistry of the artificial seawater media.

Seawater	Alkalinity (μmol kg^{-1})	DIC (μmol kg^{-1})	H^+ (NBS scale)	pH buffer capacity (mM NaOH/HCl)
2.0 mM $NaHCO_3$ pH 7.80	2183.3	2059.3	1.34×10^{-8}	0.33
2.0 mM $NaHCO_3$ pH 8.12	2309.3	2074.5	7.96×10^{-9}	0.51
2.0 mM $NaHCO_3$ pH 8.40	2493.5	2099.3	4.56×10^{-9}	0.76
0.0 mM $NaHCO_3$ pH 8.00	144.8	81.2	1.07×10^{-8}	0.02
0.5 mM $NaHCO_3$ pH 8.03	671.0	584.9	1.12×10^{-8}	0.11
1.0 mM $NaHCO_3$ pH 8.00	1180.7	1083.6	1.26×10^{-8}	0.18
2.0 mM $NaHCO_3$ pH 8.00	2206.9	2043.5	1.07×10^{-8}	0.40

Note, the alkalinity and dissolved inorganic carbon DIC were measured using a Total Alkalinity Titrator AS-ALK2 and a Dissolved Inorganic Carbon Analyzer AS-C3 (Humphreys et al. 2019) (Apollo SciTech Inc., USA), respectively; the results were calibrated using measurements of the batch 1/21 of GEOMAR/ICOS-OTC sub-standard for DIC and alkalinity. The H^+ concentrations were calculated using CO2sys_v2.1.xls, and the salinity of the seawater was 34.0‰.

Table S2. Parameters of the NICA-Donnan model used for the calculation of Fe speciation.

Proton binding constants (Lodeiro et al. 2020)	
b = 0.57, p1 = 0.59, p2 = 0.70	
Qmax1,H = 2.52, log $\tilde{K}_{H1}$ = 2.34, m_1 = 0.38	
Qmax2,H = 0.80, log $\tilde{K}_{H2}$ = 8.6, m_2 = 0.53	
Fe(III) NICA constants (Zhu et al. 2021)	
DOM1 (carboxylic-type groups)	
$\log K_{FeDOM1}$	2.94 ± 0.19
n_{FeDOM1}	0.32 ± 0.00
DOM2 (phenolic-type groups)	
$\log K_{FeDOM2}$	9.60 ± 0.00
n_{FeDOM2}	0.30 ± 0.02

Note, the modelling considers potential formation of the solid $Fe(OH)_3$. For the speciation modelling, we additionally included the model siderophore (i.e., desferrioxamine B), given for their importance in Fe chemistry and ubiquitous distribution of siderophores in surface oceans. Proton & major ions (i.e. Ca^{2+} and Mg^{2+}) binding constants of the desferrioxamine B were from (Schijf and Burns 2016), and Fe binding constants from(Butler and Theisen 2010). Major salt concentrations are the same as those used for AQUIL (Sunda, Price, and Morel 2005).

Table S3. A summary of the measured phycosphere pH in a diversity of phytoplankton species. Our pH-sensing nano-probes can work on a single cell of several µm.

Phytoplankton	Species	Cell size* (µm)	Increase of pH over bulk seawater		Method	Light	Seawater		Reference
			mean	SD			pH	HCO_3^- (mM)	
Diatom	*Coscinodiscus wailesii*	100	0.15	0.20	Nano-probe	140	8.0	2.0	This study
Green alga	*Chlamydomonas concordia*	5	0.11	0.07	Nano-probe	140	8.0	2.0	This study
Diatom	*Coscinodiscus radiatus*	50	0.41	0.04	Nano-probe	140	8.0	1.0	This study
Coccolithophore	*Emiliania huxleyi*	5	0.20	0.09	Nano-probe	140	8.0	2.0	This study
Diatom	*Odontella sinensis*	150–250	0.35		Microelectrode	200	8.0	2.0	Chrachri et al., 2018
Diatom	*Coscinodiscus sp.*	140–170	0.35		Microelectrode	200	8.0	2.0	Chrachri et al., 2018
Diatom	*Odontella mobiliensis*	40–60	0.10		Microelectrode	200	8.0	2.0	Chrachri et al., 2018
Diatom	*Thalassiosira weissflogii*	20–25	0.05		Microelectrode	200	8.0	2.0	Chrachri et al., 2018
Diatom	*Thalassiosira weissflogii*	12–22	0.04		Fluorescence dye	160	7.9	2.4	Milligan et al., 2009
Diatom	*Thalassiosira weissflogii*	12–22	0.35		Fluorescence dye	160	8.6	2.4	Milligan et al., 2009
Diatom	*Coscinodiscus wailesii*	400	0.90		Microelectrode	170	7.7	2.0	Kühn & Raven, 2008
Cyanobacteria	*Trichodesmium*	200-1600	0.30		Microelectrode	1000	8.1	2.0	Eichner et al., 2017
Cyanobacteria	*Trichodesmium*	200-1600	0.20		Microelectrode	1000	7.8	2.0	Eichner et al., 2017
Diatom	*Coscinodiscus granii*	50–60	0.29		Microelectrode	165	8.0	2.0	Kühn & Köhler-Rink, 2008
Diatom	*Coscinodiscus granii*	50–60	0.21		Microelectrode	165	8.1	2.0	Kühn & Köhler-Rink, 2008
Diatom	*Coscinodiscus granii*	50–60	0.26		Microelectrode	165	8.2	2.0	Kühn & Köhler-Rink, 2008
Chromist algae	*Phaeocystis*	1400	0.03		Microelectrode	160	8.5	NA	Ploug et al., 1999
Chromist algae	*Phaeocystis*	1400	0.30		Microelectrode	130	8.2	NA	Ploug et al., 1999

*Cell diameter or length. NA, not available. In this study, the distance between the measurement points and cell wall of the cells were roughly equal to the radius of the nano-probes (i.e., ~100 nm). The biological replicates: *n* = 32 for *Coscinodiscus wailesii*, *n* = 7 for *Chlamydomonas concordia*, *n* = 3 for *Coscinodiscus radiatus*, and *n* = 5 for *Emiliania huxleyi*. Phycosphere pH varied with ambient bicarbonate concentration, and only data for 2.0 mM bicarbonate are shown here. Unit of light, µmol m^{-2} s^{-1}.

References

Butler A, Theisen RM. Iron(III)–siderophore coordination chemistry: Reactivity of marine siderophores. Coord Chem Rev. 2010;254:288–96.

Chrachri A, Hopkinson BM, Flynn K, Brownlee C, Wheeler GL. Dynamic changes in carbonate chemistry in the microenvironment around single marine phytoplankton cells. Nat Commun. 2018;9:74.

Eichner MJ, Klawonn I, Wilson ST, Littmann S, Whitehouse MJ, Church MJ, et al. Chemical microenvironments and single-cell carbon and nitrogen uptake in field-collected colonies of *Trichodesmium* under different pCO_2. ISME J. 2017;11:1305–17.

Humphreys MP, Achterberg EP, Hopkins JE, Chowdhury MZH, Griffiths AM, Hartman SE, et al. Mechanisms for a nutrient-conserving carbon pump in a seasonally stratified, temperate continental shelf sea. Prog Oceanogr. 2019;177:101961.

Kühn SF, Köhler-Rink S. pH effect on the susceptibility to parasitoid infection in the marine diatom *Coscinodiscus* spp. (Bacillariophyceae). Mar Biol. 2008;154:109–16.

Kühn SF, Raven JA. Photosynthetic oscillation in individual cells of the marine diatom *Coscinodiscus wailesii* (Bacillariophyceae) revealed by microsensor measurements. Photosynth Res. 2008;95:37–44.

Lodeiro P, Rey-Castro C, David C, Achterberg EP, Puy J, Gledhill M. Acid-base properties of dissolved organic matter extracted from the marine environment. Sci Total Environ. 2020;729:138437.

Milligan AJ, Mioni CE, Morel FMM. Response of cell surface pH to pCO_2 and iron limitation in the marine diatom *Thalassiosira weissflogii*. Mar Chem. 2009;114:31–6.

Ploug H, Stolte W, Epping EHG, Jørgensen BB. Diffusive boundary layers, photosynthesis, and respiration of the colony-forming plankton algae, *Phaeocystis* sp. Limnol Oceanogr. 1999;44:1949-58.

Schijf J, Burns SM. Determination of the side-reaction coefficient of desferrioxamine B in trace-metal-free seawater. Front Mar Sci. 2016;3:117.

Sunda WG, Price NM, Morel FM. Trace metal ion buffers and their use in culture studies. In: Andersen RA (ed). Algal Culturing Techniques. Academic Press, Burlington, MA. 2005. pp 35-63.

Zhang Y, Takahashi Y, Hong SP, Liu F, Bednarska J, Goff PS, et al. High-resolution label-free 3D mapping of extracellular pH of single living cells. Nat Commun. 2019;10:5610.

Zhu K, Hopwood MJ, Groenenberg JE, Engel A, Achterberg EP, Gledhill M. Influence of pH and dissolved organic matter on iron speciation and apparent iron solubility in the Peruvian shelf and slope region. Environ Sci Technol. 2021;55:9372–83.
